# A Bioinspired, Multimodal Soft Tactile Skin with Task‐Adaptive Perception for Intelligent Robotic Manipulation

**DOI:** 10.1002/advs.77230

**Published:** 2026-08-18

**Authors:** Yu‐Jin Lee, Hyeonung Kang, Ju Yeon Woo, Daekyum Kim, Chang‐Soo Han

**Affiliations:** ^1^ School of Mechanical Engineering Korea University Seoul Republic of Korea; ^2^ Somatosensory Molecular Biomimetics Research Center Korea University Seoul Republic of Korea; ^3^ School of Smart Mobility Korea University Seoul Republic of Korea; ^4^ IDT Inc Hwaseong Gyeonggi Republic of Korea

**Keywords:** contribution, mechanoreceptor, multimodal sensing, tactile sensor

## Abstract

Achieving human‐like tactile perception is essential for robotic systems to perform fine manipulation and adapt to dynamic environments. However, most tactile sensors have deviated from the physiological encoding based on human mechanoreceptors. Here, we present a layered trimodal tactile sensing system that mimics Meissner, Merkel, and Ruffini receptors. These sensors can accurately distinguish stimuli (static normal pressure, dynamic shear vibration, static horizontal strain) coming from different directions and in different shapes. Using a receptor‐level analysis framework based on random forest feature importance, we systematically analyze the involvement and relative contribution of biomimetic sensors across four tactile tasks (Braille reading, texture identification, softness classification, slip detection). Notably, the cooperation of two or three sensors significantly enhances recognition accuracy for the tasks. When the sensors are integrated on a robotic gripper, they enables real‐time slip/drop detection with 97.62% accuracy and supports closed‐loop grip‐force adaptation, thereby demonstrating stabilized grip of the object under increasing load. These results help identify appropriate sensor combinations for specific tactile tasks and demonstrate the potential of the proposed system for applications in humanoid robotics, prosthetics, haptic devices, and augmented and virtual reality systems.

## Graphical abstract

Perception in robotic manipulation improves through the cooperative and complementary operation of biologically inspired multimodal tactile sensors. Receptor‐specific contribution analysis identifies which sensing modalities are required for specific tactile tasks. The results demonstrate that artificial mechanoreceptor pathways operate in a task‐adaptive manner, enabling rational sensor selection and adaptive slip control.

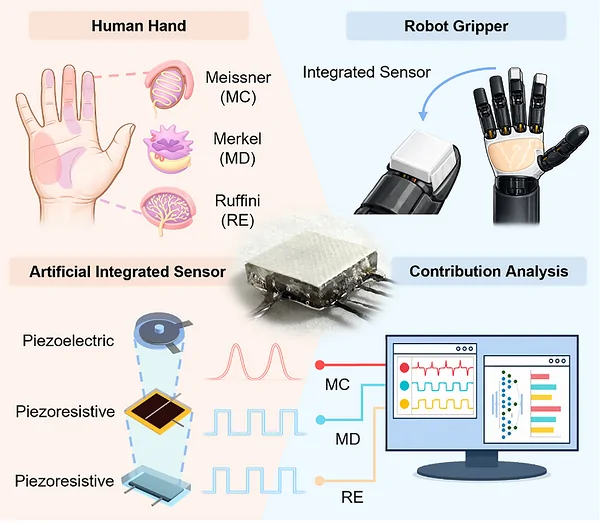

## 1 Introduction

Human tactile perception relies on four major types of cutaneous mechanoreceptors distributed across the skin, including Merkel disks, Meissner corpuscles, Ruffini endings, and Pacinian corpuscles. These receptors are generally classified into two slowly adapting (SA) receptors—SAI Merkel disks and SAII Ruffini endings—and two rapidly adapting (RA) receptors—RAI Meissner corpuscles and RAII Pacinian corpuscles. In this work, we focus on three of these receptor pathways—Merkel disks, Meissner corpuscles, and Ruffini endings—as they are closely associated with the contact‐level mechanical cues targeted here, including normal pressure, shear/slip‐related stimuli, and stretch [1, 2, 3, 4, 5].

Mechanoreceptors act cooperatively and complementarily, with their relative contributions varying by context [6]. Through such cooperative sensing, humans can discriminate surface textures, detect early signs of slippage, and immediately adjust grip force [2, 7, 8, 9]. For example, the onset of slippage involves not only changes in pressure but also shear forces and micro‐vibrations [10, 11], and the perception of softness is more strongly associated with pressure and skin stretch [12, 13, 14, 15]. Such integrated perception of complex stimuli is a key mechanism that enables stable and precise manipulation beyond simple tactile detection [16].

In contrast, most tactile sensors used in robot grippers and hands are specialized for a single physical quantity, such as pressure or stretch, and therefore do not reflect the layered distribution and functional diversity of biological mechanoreceptors [17, 18, 19, 20, 21, 22, 23]. Their restricted sensing modality also limits the ability to capture the temporal characteristics of SA and RA receptors, and this reduces sensitivity to multimodal and dynamic tactile events [24]. This constraint hinders robotic functions that rely on distinct tactile cues, including accurate contact‐state estimation and timely slip detection [25]. In this paper, we demonstrate that achieving human‐level tactile perception requires not only mimicking basic signal patterns but also incorporating the structural and functional distribution of skin and quantitatively modeling receptor‐specific contributions under multimodal conditions.

To overcome such limitations, recent studies have introduced bioinspired multimodal tactile sensors aimed at replicating human skin‐like functionality [26, 27, 28, 29, 30, 31, 32, 33, 34, 35, 36], showing potential for use in robotic grippers and hands. For instance, hybrid sensors combining triboelectric and piezoresistive mechanisms have been developed to reproduce both SA and RA responses [26]. Another study integrated artificial receptors mimicking Meissner, Merkel, and Ruffini receptors to improve texture classification and Braille recognition performance [34]. While these approaches show the advantages of multimodal sensing, a fundamental gap still remains in quantitatively identifying which mechanoreceptor pathways dominate under complex tactile stimuli and how each pathway contributes to tactile perception. Addressing this gap requires not only integrating multiple sensing elements but also clarifying their receptor‐specific and task‐dependent functional contributions.

Consequently, in the absence of such receptor‐specific analysis, mechanoreceptor‐inspired tactile sensing systems often rely on redundant sensing modalities or high‐dimensional learning pipelines, which increases system complexity and makes real‐time, task‐responsive robot manipulation more difficult. This gap limits the translation of mechanoreceptor principles into robotic tactile sensing systems, particularly for robotic manipulators performing repetitive or task‐specific operations [37, 38]. For example, in precise grasping, slip detection requires rapid tactile feedback to be integrated within tight control loops for timely grip‐force modulation. In such scenarios, RA pathways are primarily responsible for detecting incipient slip events, whereas SA pathways primarily encode sustained contact forces [25, 39]. Without quantitative separation of these contributions, learning‐based manipulation systems must infer task‐relevant tactile structure implicitly from high‐dimensional inputs, which increases training complexity and hinders task‐specific generalization [40, 41, 42]. For instance, when a robotic hand grasps an object while stimultaneously experiencing shear vibration, rough surface texture, and deformation, most multimodal sensors have difficulty independently discriminating each stimulus and accurately identifying the object state.

In this study, we propose a biomimetic trimodal tactile sensor system that integrates three types of artificial receptors—mimicking Meissner, Merkel, and Ruffini receptors—into a skin‐inspired layered structure that can be attached to robotic grippers (Figure 1A). With this sensor, we quantify each artificial receptor's contribution to four tactile perception tasks, including Braille reading, texture recognition, slip detection, and softness perception, revealing their task‐dependent and complementary roles in processing task‐relevant multimodal tactile cues (Figure 1B). When mounted on a robotic gripper (Figure 1C), the system provides intuitive real‐time slip feedback based on distinct receptor‐like outputs and supports dynamic manipulation without relying on a complex inference algorithm. We also validated its industrial potential by successfully distinguishing automotive surface textures. The receptor‐specific contribution analysis presented in this study provides a quantitative basis for identifying task‐relevant receptor pathways and their complementary roles, thereby reducing system complexity in human‐like tactile sensing and enabling task‐optimized robotic manipulation [43, 44].

**FIGURE 1:**
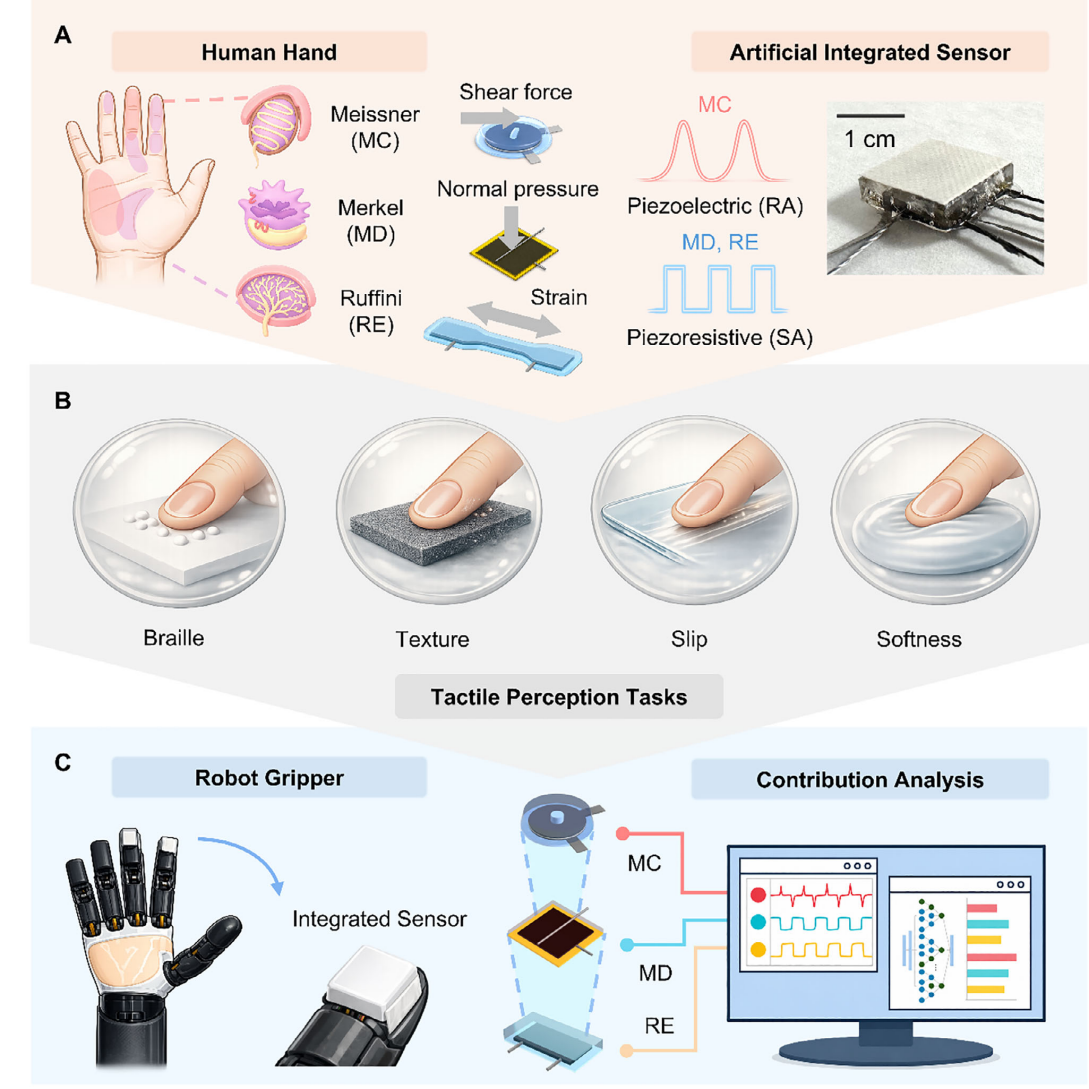
Bioinspired trimodal tactile sensor system for multimodal perception and receptor‐contribution analysis. (A) Biological inspiration and artificial sensor design. Representative cutaneous mechanoreceptors—Meissner corpuscles, Merkel disks, and Ruffini endings—and their corresponding artificial sensing units designed to detect shear force, normal pressure, and strain, respectively, through piezoelectric and piezoresistive sensing mechanisms; photograph of the fabricated integrated sensor. (B) Representative perceptual tasks enabled by the integrated sensor: Braille recognition, surface texture recognition, slip detection, and softness recognition. (C) Integration of the trimodal tactile sensor onto a robotic gripper and schematic illustration of task‐dependent receptor‐contribution analysis for multimodal tactile interpretation.

## 2 Results

### 2.1 Receptor‐Specific Response Characteristics of the Integrated Sensor

The integrated multimodal tactile sensor is designed to replicate the physiological and functional characteristics of Meissner corpuscles, Merkel disks, and Ruffini endings found in human skin (Figures S1 and S5). Each receptor‐inspired unit exhibits preferential rapidly adapting (RA) or slowly adapting (SA) response characteristics, while the three channels retain distinguishable and complementary signal patterns under complex mechanical loading despite limited mechanical cross‐coupling (Figure 1A and Figures S11 and S12).

The artificial Meissner sensor is a piezoelectric device inspired by RA‐type mechanoreceptors and can detect fine vibrations and shear forces [2, 6, 7, 45]. It is designed to respond predominantly to lateral vibration rather than normal vibration (Figure S2 and Table S2). In contrast, the artificial Merkel and Ruffini sensors emulate SA‐type mechanoreceptors and operate via a piezoresistive mechanism to detect static pressure and strain, respectively [2, 7, 46]. These sensors are highly sensitive to the pressure in the normal direction and strain in the horizontal direction, respectively (Figures S3 and S4), but are less sensitive to the stimuli in different directions. The resulting resistance changes are converted into voltage outputs using a custom voltage‐divider circuit (see Movie S1 and Figure S27).

The three receptor‐inspired sensor units were vertically stacked to reflect the physiological distribution of mechanoreceptors in human skin and encapsulated with polydimethylsiloxane (PDMS) – which serves as an artificial epidermal layer – to form an integrated multimodal tactile sensing module. A microscale‐patterned high‐friction film was laminated onto the surface to enhance robustness and contact stability, like a fingerprint, enabling reliable multimodal tactile sensing under complex mechanical interactions. Detailed fabrication procedures are described in the Materials and Methods section. We first evaluated the electrical responses of the integrated sensor under mechanical loading, which allowed us to verify how each receptor‐inspired sensor reproduces the physiological characteristics of its biological counterpart—Meissner, Merkel, and Ruffini receptors.

We then examined how indentation speed (1, 5, 10, and 15 mm/s) affects the sensor outputs under a constant normal load of 4 N (Figure 2A). Faster indentation increased the Meissner output substantially, whereas the Merkel and Ruffini signals remained nearly unchanged (Figure 2D). This behavior reflects the RA‐type nature of the Meissner sensor, which responds strongly to rapid changes in the stimulus. Conversely, the SA‐type Merkel and Ruffini sensors, which respond to steady or slowly varying loads, exhibited minimal sensitivity to loading speed. These results show that while the Meissner sensor is primarily tuned for vibration and shear detection, it also responds to rapid normal loading because fast impacts generate lateral deformation within the layered structure.

**FIGURE 2:**
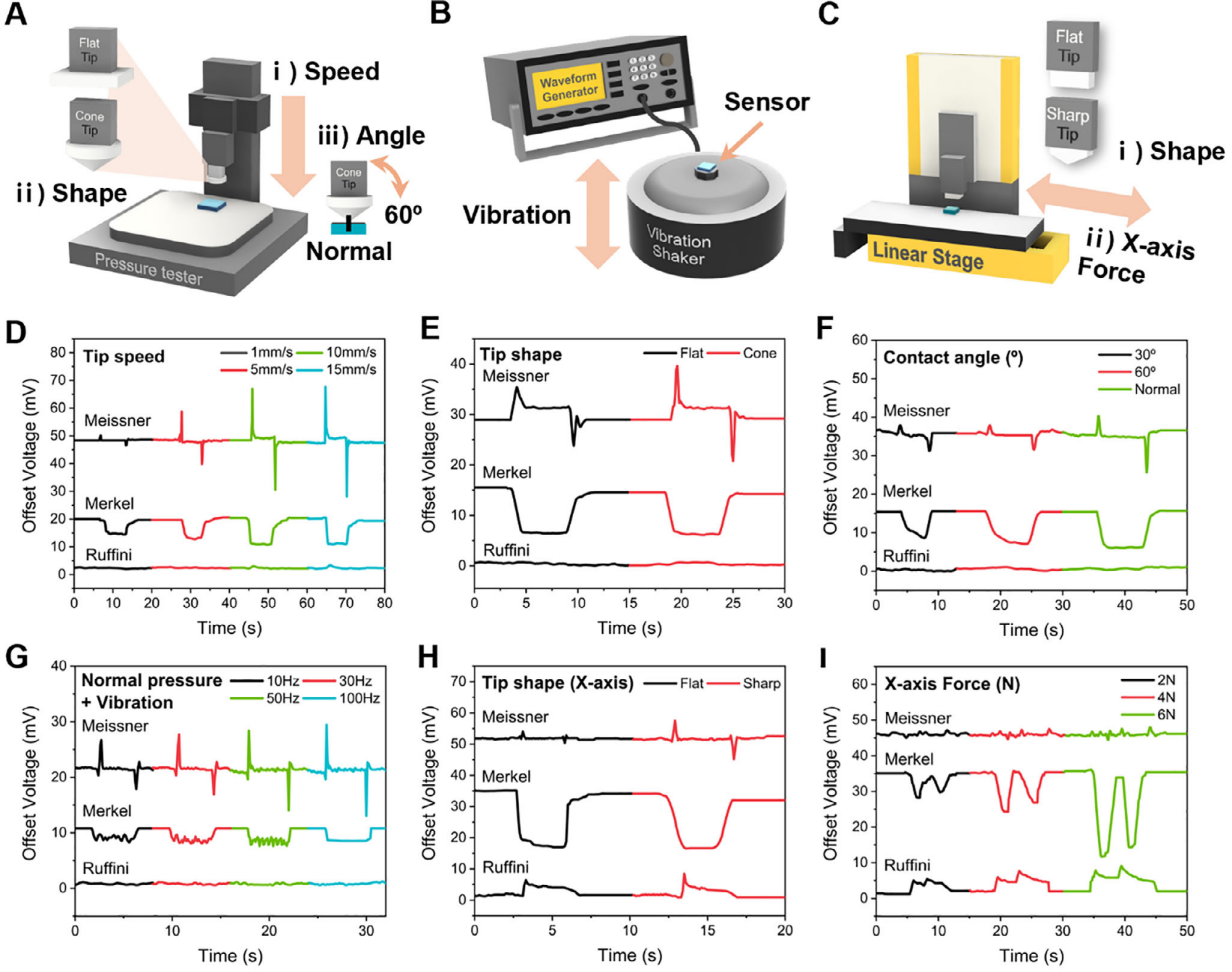
Characterization of the integrated bioinspired tactile sensor under diverse mechanical stimuli. (A) Normal‐loading setup with controlled tip speed (1, 5, 10, and 15 mm/s), geometry (conical or flat), and contact angle (0°, 30°, and 60°). (B) Combined pressure–vibration setup with vertical oscillations at 10, 30, 50, and 100 Hz. (C) Lateral‐loading setup in which an indenter is translated along the *x*‐axis using a linear stage. (D) Sensor responses under a constant normal load of 4 N using a flat indenter at 0° and speeds of 1, 5, 10, and 15 mm/s. (E) Responses to flat and conical indenters under identical normal loading. (F) Sensor responses under the same normal load at contact angles of 0°, 30°, and 60°, where 0° corresponds to vertical loading along the surface‐normal direction. (G) Sensor response to combined pressure and vibration at 10–100 Hz, illustrating dynamic frequency–pressure interaction. (H) Lateral‐sliding responses obtained with flat and sharp tips at 25 mm/s. (I) Responses to reciprocating shear loads of 2, 4, and 6 N applied along the *x*‐axis.

Next, we explored how different contact geometries—cone and flat tips—affect the sensor response under the same loading conditions as the first experiment (Figure 2A). Each tip was attached to the actuator and pressed against the sensor surface. The cone tip generated localized stress and steep lateral deformation, eliciting a pronounced Meissner response (Figure 2E). In contrast, the Merkel sensor showed little variation because it mainly responds to steady, uniformly distributed normal pressure, while the Ruffini sensor remained inactive owing to the absence of tensile strain.

We also examined how the contact angle of the stimulus affects sensor responses. A flat indenter was tilted to contact angles of 0°, 30°, and 60°, defined as the angle between the indenter axis and the surface‐normal direction; thus, 0° corresponded to vertical loading (Figure 2A). Vertical loading (0°) produced immediate voltage responses, whereas oblique contacts generated delayed signals in the Merkel sensor (Figure 2F). This is because inclined contact begins with a smaller contact area, reducing transmitted force and slowing pressure buildup.

To assess frequency‐dependent behavior, we applied sinusoidal vibration (10–100 Hz) using an electrodynamic shaker while maintaining static pressure with a 200 g weight on top of the sensor (Figure 2B). The Merkel sensor exhibited a clear response to the applied normal preload and also showed detectable variations under low‐frequency vibration because the vibration periodically modulated the normal contact force. However, its vibration‐related response became substantially weaker at higher frequencies, including 100 Hz (Figure 2G). This frequency‐dependent behavior closely resembles that of biological Merkel disks [46], demonstrating that the artificial sensor effectively reproduces their SA characteristics. The Meissner sensor exhibited selective responses to vibration rather than normal pressure when both stimuli were simultaneously applied, consistent with the behavior of biological RA‐I mechanoreceptors.

Finally, we evaluated the response to lateral shear force using a linear motor (Figure 2C). A sharp tip was first brought into contact with the sensor surface and then translated laterally to apply shear before being released, allowing the responses during the contact–sliding–release sequence to be monitored. Under sharp‐tip contact, the Meissner and Ruffini sensors exhibited stronger shear‐related responses than under flat‐tip contact. In contrast, flat‐tip contact produced a markedly reduced Meissner response, consistent with the lower lateral stress distributed across a wider contact area (Figure 2H). We then applied reciprocating shear loads of 2, 4, and 6 N along the *x*‐axis using a flat tip. One forward‐and‐backward cycle was performed at each force level; therefore, the two consecutive signal responses represent the forward and backward motions, respectively. As the applied shear force increased, the response amplitudes of the three sensing channels also increased (Figure 2I). Additional x‐ and y‐direction shear tests further confirmed that the integrated sensor could detect shear‐related events in both in‐plane directions, although the present single‐unit design was intended for rapid shear‐event detection rather than precise discrimination of the absolute shear direction (Figure S9). These results confirm that the integrated tactile sensor can detect lateral shear events under different contact conditions, distinguish contact‐induced stress distributions, and provide a quantitative response proportional to the applied load. Additional response‐time measurements further confirmed that the three artificial tactile sensing elements generated rapid and repeatable transient responses under their corresponding mechanical stimuli, supporting their suitability for real‐time tactile sensing applications (Figure S10).

Overall, these characterization results confirmed that the three biomimetic sensing channels exhibited preferential responses to dynamic shear and vibration, normal pressure, and tensile deformation, respectively, under diverse loading conditions (Figure 2D–I).

### 2.2 Spatial Pattern Discrimination with Braille and Texture Tasks

Tactile spatial pattern recognition requires resolving localized contact features arising from surface protrusions, texture, and frictional interactions. To evaluate this capability, two representative tactile tasks—Braille recognition and surface‐texture classification—were investigated in this study.

Braille recognition represents a high‐resolution tactile task in which arrays of raised dots must be accurately detected within a confined area. Although Braille perception is fundamentally based on static stimuli, active exploration inevitably introduces dynamic components, including shear‐force modulation and frictional variations during scanning. In this study, the word “TACTILE” was fabricated in Braille format using 3D printing (Figure S13). An integrated trimodal tactile sensor was mounted on the index fingertip, and Braille characters were scanned sequentially from top to bottom following the standard 2 × 3 Braille cell structure (Figure 3A). During scanning, the three mechanoreceptor‐mimetic sensors exhibited distinct signal characteristics. The Merkel sensor produced clear responses to localized pressure changes generated by individual Braille dots, whereas the Meissner sensor responded to edge contact during dot scanning, indicating the occurrence of shear vibration. The Ruffini sensor primarily reflected gradual strain variations associated with global finger deformation during the scanning process.

**FIGURE 3:**
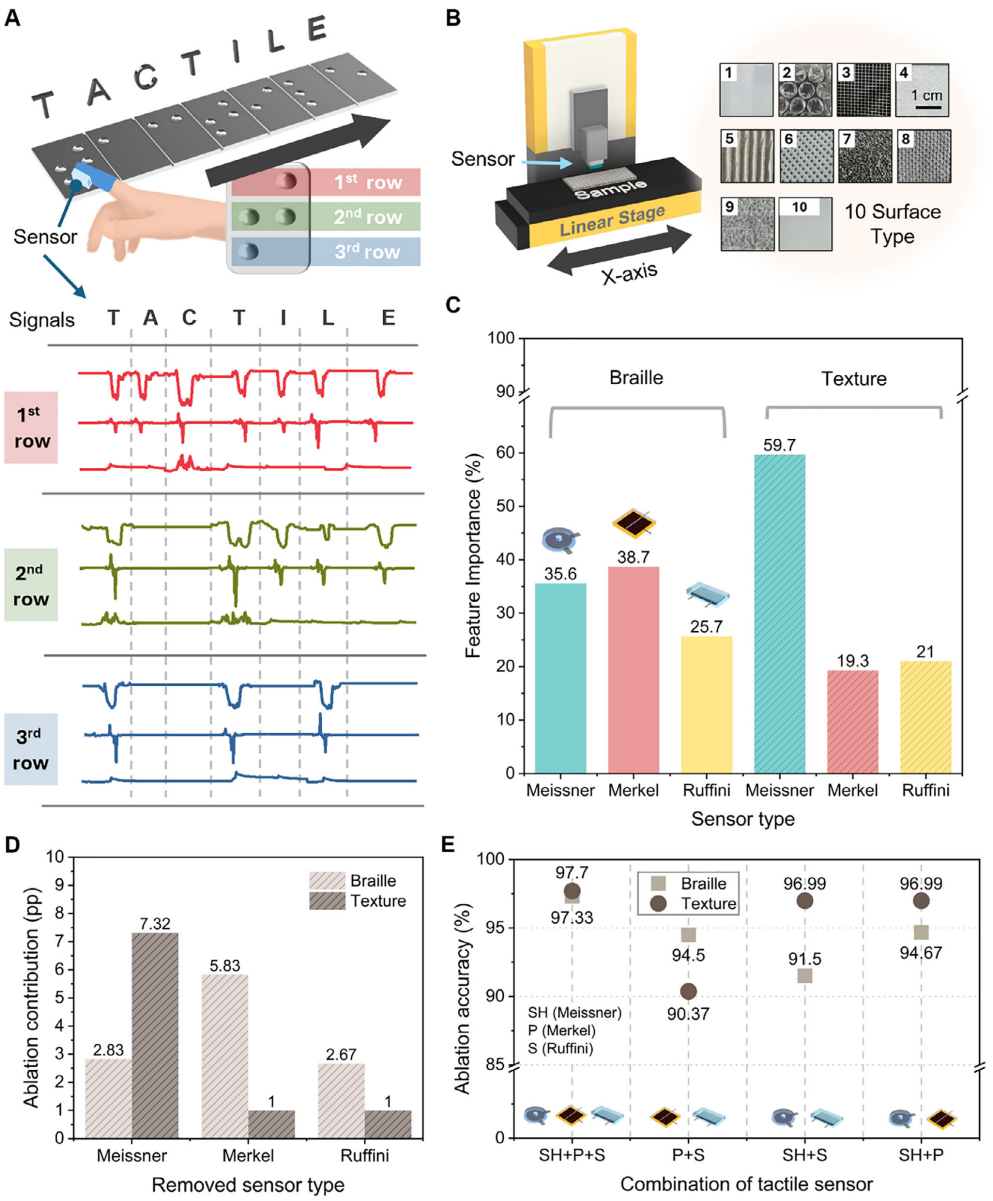
Braille and texture recognition via an integrated bioinspired tactile sensor: sensor contribution analysis. (A) Schematic of the Braille reading task and corresponding signal acquisition from the receptor‐mimetic sensor units—Merkel, Meissner, and Ruffini (top to bottom)—as the sensor mounted on a human finger scans the word “TACTILE” in Braille line by line. (B) Surface texture classification setup and tested materials, numbered from 1 to 10: rubber, bubble wrap, stainless steel mesh, glass, corrugated cardboard, nonwoven, sandpaper, fabric, foam, and paper. (C) Feature importance results showing the contribution of each sensor type in Braille recognition and texture classification. (D) Ablation contribution results showing the performance drop when each sensor type is removed in Braille and texture tasks. (E) Classification accuracy obtained from different sensor combinations in the ablation study.

In addition, a surface‐texture classification experiment was conducted to evaluate tactile perception during dynamic exploration (see Movie S2 and Figure S14). Ten representative materials—rubber, bubble wrap, stainless steel mesh, glass, corrugated cardboard, nonwoven fabric, sandpaper, fabric, foam, and paper—were selected to represent a wide range of surface structures and frictional characteristics (Figure 3B).

To quantitatively analyze the functional contributions of the mechanoreceptor‐inspired sensing modalities, two complementary approaches were employed: feature‐importance analysis and ablation‐based contribution analysis. Random Forest‐based feature importance reflects how much each sensor signal contributes to improving the predictive performance within the Random Forest classifier, representing the relative importance of each sensing modality in the model. In contrast, ablation contribution measures the decrease in classification accuracy when a specific sensing modality is removed, thereby quantifying the functional necessity of that sensing modality for the task.

Random Forest‐based feature‐importance analysis revealed task‐dependent differences in sensor contributions (Figure 3C). In Braille recognition, the Merkel sensor exhibited the highest contribution (38.7%), followed by the Meissner (35.6%) and Ruffini (25.7%) sensors. This indicates that sustained pressure signals and localized contact cues play a dominant role in distinguishing Braille dot patterns. In contrast, texture classification showed a markedly different contribution pattern, in which the Meissner sensor dominated the task with a contribution of 59.7%, while the Merkel and Ruffini sensors contributed 19.3% and 21.0%, respectively.

Ablation analysis using the representative Random Forest classifier further confirmed this trend (Figure 3D). For Braille recognition, removing the Merkel sensor resulted in the largest performance decrease (5.83 percentage points, pp), whereas removing the Meissner and Ruffini sensors reduced accuracy by 2.83 and 2.67 pp, respectively. This indicates that pressure‐based sensing provides the most critical information for identifying Braille dot patterns. In contrast, for texture classification, removing the Meissner sensor caused the largest accuracy drop (7.32 pp), while removing either the Merkel or Ruffini sensors reduced accuracy by only about 1 pp.

The highest classification accuracy was achieved when all three sensing modalities were fully integrated (Figure 3E), reaching 97.33% for Braille recognition and 97.7% for texture classification. Notably, removing the shear sensor caused the most significant performance degradation in the texture task, whereas removing the pressure sensor most strongly affected Braille recognition.

Taken together, the results of Braille recognition and texture classification reveal a clear functional specialization among the mechanoreceptor‐mimetic sensors. Pressure‐sensitive Merkel sensors play a dominant role in spatial pattern recognition, whereas shear‐sensitive Meissner sensors primarily govern surface‐texture discrimination. The predominant role of the Meissner sensor can be attributed to its high sensitivity to dynamic shear forces and lateral surface deformation—key cues for detecting subtle changes in surface roughness. This observation aligns well with psychophysical evidence suggesting that human texture perception primarily relies on differentiating micro‐scale shear cues generated during surface exploration [25].

Furthermore, the ablation analysis demonstrates that the dominant mechanoreceptive pathway varies depending on the tactile task. This division of roles mirrors the functional specialization of human mechanoreceptors, particularly the Type‐I Merkel and Meissner pathways concentrated near the epidermal–dermal boundary, which are known to play critical roles in fine tactile perception [25, 47]. To verify that these receptor‐contribution trends were not specific to the Random Forest classifier, additional multi‐classifier ablation analysis was performed, and consistent task‐dependent trends were observed across different classifiers (Figure S23). Additional Random Forest‐based confusion matrices for single‐ and multimodal sensor combinations in Braille recognition and texture classification are provided in the Supporting Information, further supporting the modality‐dependent classification trends observed in the main results (Figures S18 and S19).

### 2.3 Dynamic Tactile Interaction Analysis for Slip and Softness

Slip detection is critical for performing stable object manipulation [48, 49], requiring rapid identification of subtle shear and force variations at the contact interface. A two‐finger robotic gripper equipped with the integrated tactile sensor was used (Figures S8 and S15). The gripper held a 30‐mL vial, and gradual slippage was induced by incrementally attaching additional weight to the vial. In a separate grasp–lift–hold–release manipulation test, the three mechanoreceptor‐mimetic sensors exhibited distinct phase‐dependent activation patterns during object handling, further validating their complementary roles in robotic grasping (Figure S24).

Signals from the Merkel, Meissner, and Ruffini sensors exhibited distinct temporal patterns across grasping, slip onset, and object drop (Figure 4A). Upon initial contact, the Merkel channel encoded normal‐pressure changes, while the Meissner channel produced a transient voltage peak driven by micro‐vibrations. The Ruffini sensor showed minimal activity during the initial grip phase but became responsive when slippage began as strain developed within the gripper surface. As the vial accelerated downward and eventually dropped, the Ruffini signal exhibited another pronounced change, reflecting the increased surface stretch. The Merkel sensor responded transiently to pressure fluctuations from the added weight, while the Meissner sensor produced distinct peaks at slip onset and release due to rapid shear‐ and vibration‐related events. After the vial detached, the Meissner sensor briefly captured the impact vibration, after which all sensor outputs returned to baseline. Additional slip responses obtained using vial surfaces with different material characteristics are provided in Figure S16.

**FIGURE 4:**
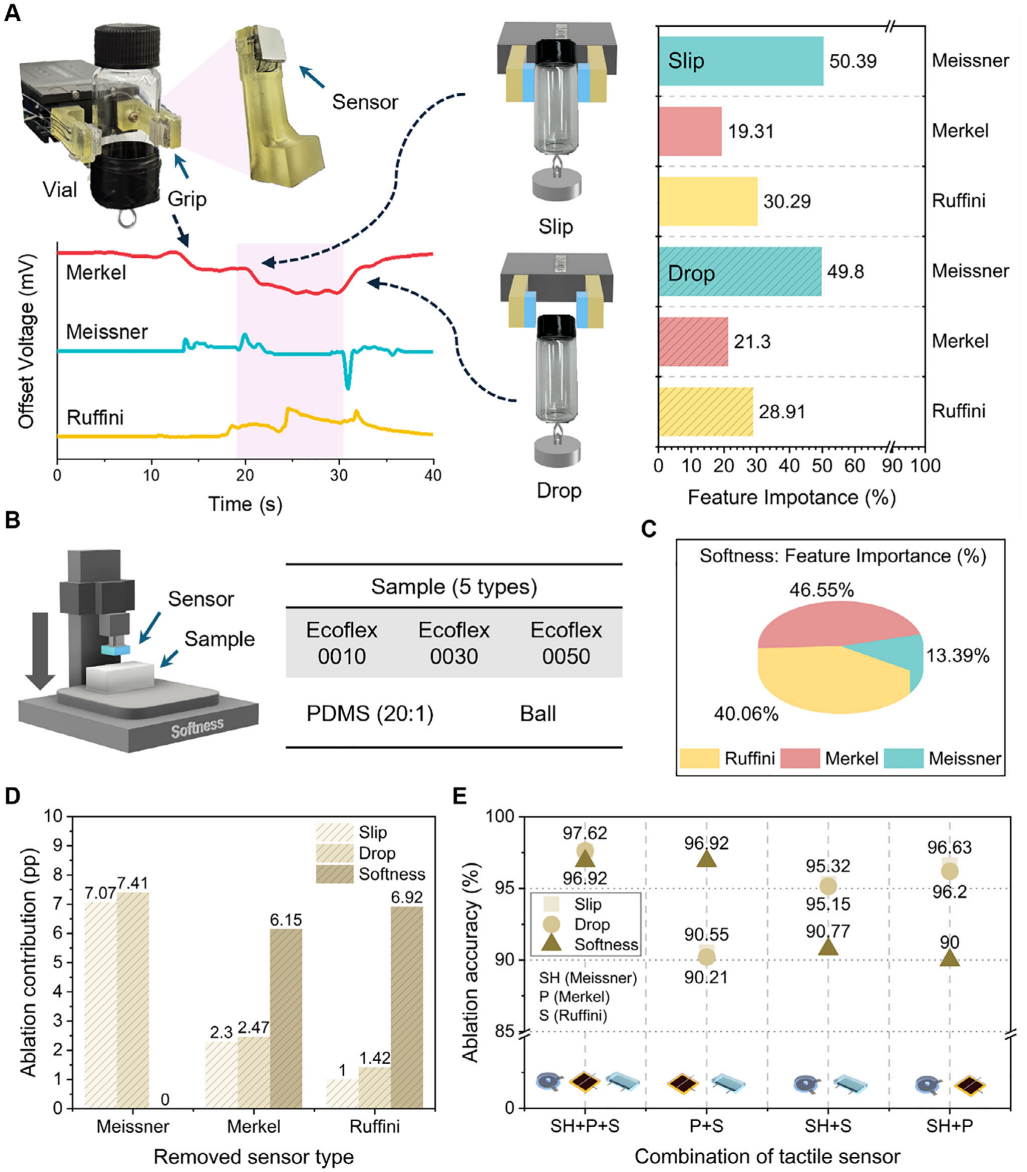
Softness and slip detection via an integrated bioinspired tactile sensor: sensor contribution analysis. (A) Slip‐detection setup using a two‐finger robotic gripper equipped with the integrated tactile sensor; representative responses from receptor‐mimetic sensors during slip events and corresponding feature‐importance analysis for slip detection and the drop event. (B) Schematic of the softness classification setup using a linear actuator with the sensor mounted at the tip, tested on samples of varying compliance: Ecoflex 0010, 0030, 0050, PDMS (20:1), and a rigid ball. The corresponding table summarizes material properties. (C) Feature importance results in softness classification, showing the relative contributions of Merkel, Meissner, and Ruffini sensors. (D) Ablation contribution results showing the performance drop when each sensor type is removed in softness and slip classification tasks. (E) Classification accuracy obtained from different sensor combinations in the ablation study.

To further evaluate tactile perception under deformation‐based interactions, softness classification experiments were performed using a linear actuator with the sensor mounted at the probe tip (see Movie S3 and Figure S17). Five materials with different compliance levels—Ecoflex 0010, Ecoflex 0030, Ecoflex 0050, PDMS (20:1), and a rigid ball—were tested to represent a wide range of mechanical stiffness (Figure 4B).

Random Forest‐based feature‐importance analysis revealed that the Meissner sensor contributed most strongly to slip‐state recognition, accounting for 50.39% of the importance for slip detection and 49.80% for drop detection (Figure 4A). This dominant contribution reflects the high sensitivity of the Meissner sensor to dynamic shear forces and micro‐vibrations generated during slip onset and object release. In contrast, softness classification exhibited a different contribution pattern (Figure 4C). The Merkel sensor showed the largest contribution (46.55%), followed by the Ruffini sensor (40.06%), while the Meissner sensor contributed the least (13.39%). These results indicate that pressure and strain signals generated by material deformation under constant loading primarily govern softness perception.

To further evaluate the functional necessity of each sensing modality, ablation‐based contribution analysis was performed using the representative Random Forest classifier (Figure 4D). For slip detection, removing the Meissner sensor resulted in the largest performance decrease (7.07 pp), while a similar trend was observed for drop detection, where removing the Meissner sensor reduced accuracy by 7.41 pp. These results indicate that shear‐related signals play a crucial role in detecting dynamic contact transitions such as slip onset and object release. In contrast, for softness classification, removing the Merkel sensor resulted in a performance decrease of 6.15 pp, while removing the Ruffini sensor reduced accuracy by 6.92 pp. Removing the Meissner sensor, however, produced almost no change in classification accuracy.

The ablation accuracy analysis further supported this interpretation (Figure 4E). For slip and drop detection, the highest classification accuracy (97.62%) was achieved when all three sensing modalities were integrated. In contrast, for softness classification, removing the Meissner sensor yielded the same classification accuracy (96.92%) as the full three‐sensor configuration. This indicates that shear sensing provides limited information for compliance estimation, whereas pressure and strain signals constitute the dominant tactile cues for softness recognition.

Interestingly, although the Meissner sensor showed a non‐negligible feature‐importance contribution (13.39%) in softness classification, the ablation analysis revealed almost no performance degradation when the sensor was removed. This discrepancy suggests that while shear‐related features were occasionally used by the Random Forest classifier, the essential information required for compliance discrimination was already sufficiently captured by the Merkel and Ruffini sensors. Therefore, shear cues may provide auxiliary information but are not functionally essential for softness recognition under the tested experimental conditions.

Taken together, these results demonstrate task‐dependent functional specialization of the mechanoreceptor‐mimetic sensors during dynamic tactile interactions. The Meissner sensor is critical for detecting slip/drop events involving rapid shear transients and micro‐vibrations, whereas the Merkel and Ruffini sensors are more important for softness recognition based on pressure and strain responses generated by material deformation. Additional multi‐classifier ablation analysis showed consistent task‐dependent trends, confirming that these receptor‐contribution patterns were not specific to the Random Forest classifier (Figure S23). Additional Random Forest‐based confusion matrices for single‐ and multimodal sensor combinations in slip/drop detection and softness classification (Figures S20 and S21), as well as those obtained using the fully integrated trimodal sensor across all tactile tasks, are provided in the Supporting Information, further supporting the task‐dependent modality contributions and high classification performance observed in the main results (Figure S22).

### 2.4 Applications in Slip Control and Defect Detection

Tactile sensing plays a crucial role in detecting subtle surface features during object interaction. To evaluate this capability, we examined the sensor's ability to detect fine surface defects using a 1:35‐scale vehicle model that reproduces the curvature and structural complexity of real automotive components (Figure 5A). Three representative surface defects were examined: double scratch lines, a single deep scratch line, and a raised surface protrusion. During each trial, the sensor was pressed onto the surface under a constant normal load and linearly scanned across the target region. Outputs from the Meissner, Merkel, and Ruffini units were continuously recorded throughout the contact, scanning, and release phases.

**FIGURE 5:**
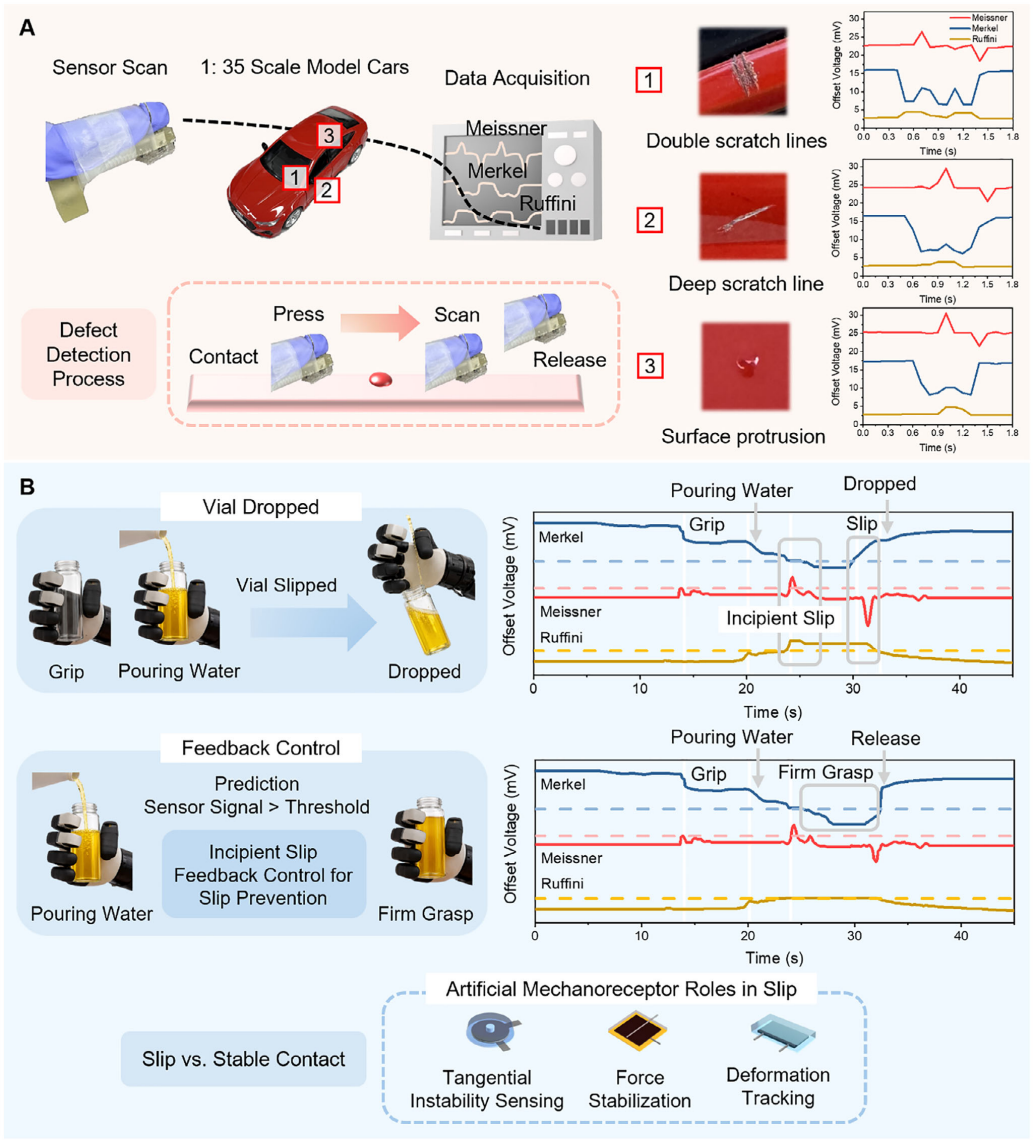
Surface scratch detection using a 1:35‐scale vehicle platform and slip‐feedback control with a prosthetic hand. (A) Surface‐defect detection using a 1:35‐scale vehicle model representing the curvature and geometric complexity of real automotive components; examined defects include double scratch lines, a deep scratch line, and a surface protrusion, with corresponding sensor responses. (B) Real‐time slip‐feedback control using a prosthetic hand equipped with the integrated tactile sensor. Incipient slip detected at t ≈ 24.0 s, identified by Meissner and Ruffini responses, triggers an increase in grip force to prevent object dropping; without feedback, the vial drops at t ≈ 31.5 s. A schematic summarizes mechanoreceptor roles in distinguishing stable contact from slip.

On defect‐free surfaces, the Meissner sensor produced a stable output of approximately 8 mV during contact and showed a transient drop to around 4 mV upon release. The Ruffini sensor detected low‐level stretch signals (∼3 mV) during scanning motion, whereas the Merkel sensor remained largely unresponsive under uniform pressure conditions, consistent with the absence of localized perturbations (Figure S25).

On the double‐scratch surface, the Merkel sensor showed clear deviations at each groove, while the Meissner sensor produced transient peaks associated with shear disturbances during probe traversal. The Ruffini sensor also exhibited noticeable voltage variations near the defect locations, reflecting disrupted strain evolution as the surface deformation was no longer smoothly distributed across the contact interface.

In the single deep‐scratch test, the Merkel sensor output briefly decreased, indicating a localized reduction in contact pressure as the probe entered the groove. The Meissner sensor responded transiently at the scratch boundaries due to shear disturbances, while the Ruffini sensor exhibited a short‐term increase in strain signal when the probe entered the depression, reflecting localized surface deformation.

For the raised surface protrusion, both Meissner and Merkel sensors exhibited pronounced responses due to increased local pressure and shear forces. The Ruffini sensor showed a distinct tensile change corresponding to the elevated feature, indicating surface stretching induced by the protrusion.

These signal patterns were consistently reproduced across multiple trials, confirming the stability and repeatability of the sensor's differential encoding mechanism. In addition, the graded responses obtained from sandpaper samples with increasing surface roughness further support the sensor's ability to encode texture severity in a quantitative manner (Figure S26). Overall, these results demonstrate that the integrated tactile sensor can effectively detect fine surface defects even on curved and geometrically complex surfaces. Its ability to identify subtle anomalies on automotive components highlights its strong potential for practical industrial inspection.

Beyond surface defect detection, tactile sensing is also essential for detecting incipient slip during object manipulation. The human hand can instantly detect slip with remarkable precision [50], and recent robotic systems have increasingly sought to replicate this capability. Prior approaches have typically relied on friction‐ or vibration‐based sensing—for example, the Southampton Hand used contact vibration to adjust grip force [51], and the BaroTac system detected slip via barometric pressure changes [52]. More recently, optical fiber‐based tactile sensors have been developed for simultaneous detection of friction, shear force, and slip [53]. However, multimodal tactile interfaces that emulate the cooperative functions of biological mechanoreceptors remain limited.

To evaluate the slip‐detection capability and feedback‐control performance of the integrated tactile sensor, experiments were conducted using a robotic hand equipped with the sensor (Figure 5B). The experiment began with the robotic hand holding an empty vial using an initial low‐grasping command. Water was then gradually poured into the vial to progressively increase the load while monitoring grasp stability.

In this study, incipient slip was defined as the unstable contact state immediately preceding full sliding or dropping, where slight oscillations, shear fluctuations, and contact instability appeared while the vial was still held by the gripper. To determine the thresholds, slip experiments were repeated under the same grasping condition, and the characteristic signal changes observed at the onset of incipient slip were used as threshold criteria.

The thresholds were defined as voltage changes relative to the initial no‐stimulus baseline of each sensing channel. Incipient slip was detected when the Artificial Merkel sensor showed a voltage decrease greater than 8 mV from its baseline, when the Artificial Meissner sensor showed a transient voltage increase greater than 4 mV from its baseline, and when the Artificial Ruffini sensor showed a voltage change greater than 3 mV from its baseline. The dashed lines in Figure 5B indicate the corresponding threshold levels for each sensing channel.

As the vial weight increased, the Artificial Meissner sensor responded to subtle shear fluctuations and micro‐vibrations, while the pressure‐based Artificial Merkel sensor showed a voltage decrease due to increased normal contact force. At the same time, accumulated deformation at the contact interface generated strain signals in the Artificial Ruffini sensor. These simultaneous responses were interpreted as a characteristic incipient‐slip tactile signature. Without feedback control, continued loading led to full sliding and eventual dropping of the vial.

When feedback control was enabled, grip correction was triggered when the multimodal slip‐precursor signals satisfied the predefined threshold criteria. The feedback control was implemented as a threshold‐based serial‐command switching scheme rather than as continuous proportional force modulation. The sensor signals were monitored in real time (see Movie S4). For the feedback‐control demonstration, the sensor voltage outputs were sampled at 100 Hz using an external microcontroller‐based interface connected to a PC. Once the predefined threshold criteria were satisfied, a gripper‐control command was transmitted to the robotic hand controller via serial communication through the COM port (see Movie S5). This command switched the gripper from the initial low‐grasping state to a stronger grasping state. Therefore, the feedback action was not implemented by adding a specific force value in newtons, but by switching between predefined gripper command states.

After the feedback command was applied, the robotic hand tightened its hold on the vial, producing a further decrease in the Artificial Merkel sensor voltage, indicating increased contact force. The Artificial Ruffini sensor exhibited minimal additional deformation, indicating stabilization of the contact interface. These results demonstrate that the Artificial Meissner, Artificial Merkel, and Artificial Ruffini sensors provide complementary shear, pressure, and strain cues for detecting slip precursors and enabling stable grasp control.

## 3 Discussion

A critical aspect that has been insufficiently addressed in prior work is the quantitative evaluation of how much—and in what manner—each mechanoreceptor pathway contributes to tactile perception under specific tasks and stimulus conditions. Although several studies have reported performance gains when integrating RA‐ and SA‐like sensors [28, 34, 54, 55, 56], they rarely provide a systematic analysis of which receptor combinations drive these improvements or during which perceptual processes such contributions emerge. Moreover, even within the SA class, the distinct and potentially complementary roles of Ruffini‐ and Merkel‐like pathways—particularly in tasks such as softness identification—have not been thoroughly investigated [14, 15, 57]. Overall, previous studies have offered limited experimental and analytical justification for why multiple mechanoreceptor pathways are necessary and how their individual functions collectively support tactile perception.

Our approach systematically evaluates how individual pathways and their cooperative interactions influence tactile perception performance under conditions in which multiple mechanoreceptors are simultaneously activated, providing a structural view consistent with the pathway‐level organization of human touch. In particular, this analysis provides practical guidance for identifying effective combinations of artificial receptors for task‐specific tactile perception. Notably, although the human brain can to some extent distinguish signals originating from different mechanoreceptor types [1, 25, 58], quantitatively separating the cooperative processes through which multiple mechanoreceptors jointly give rise to tactile perception remains challenging [6]. In this study, we successfully address this challenge by quantitatively analyzing the task‐dependent contributions of multiple mechanoreceptor‐inspired sensing pathways. This analysis provides meaningful insights into human tactile mechanisms by enabling an indirect yet quantitative assessment of mechanoreceptor‐level contributions.

From this perspective, our results can be extended to identify the functional and perceptual roles of individual mechanoreceptors during specific tactile tasks, offering a structured means of understanding human tactile perception. Beyond biomimicry, this knowledge enables the rational design of task‐specific tactile sensors optimized for robotic manipulation [37, 59]. Furthermore, incorporating a Pacinian‐like high‐frequency sensing layer may extend this framework to capture fine textures and tool–surface interactions, thereby further broadening the scope of tactile perception [60, 61, 62, 63].

Robotic manipulation relies heavily on tactile sensing to estimate contact forces, detect incipient slip, and maintain a stable grasp [16, 30, 64, 65, 66, 67]. Achieving human‐level dexterity requires interpreting diverse mechanical stimuli—such as pressure, shear, vibration, and stretch—at a receptor‐specific level, analogous to the human tactile system [37, 54, 68]. In this study, we present a bio‐inspired integrated tactile architecture in which Meissner‐type, Merkel‐type, and Ruffini‐type sensing pathways are structurally separated, and we quantitatively analyze their receptor‐level contributions across diverse tactile manipulation tasks.

In summary, this study quantitatively elucidates how complementary tactile sensing pathways contribute differentially depending on the manipulation task and proposes a receptor‐level tactile sensing framework for robotic manipulation. By structurally separating Meissner‐, Merkel‐, and Ruffini‐like pathways and analyzing their task‐dependent contributions, we move beyond indiscriminate fusion of multimodal tactile signals. In addition, our results provide clear guidance on which tactile cues should be prioritized or selectively combined for specific tasks. Ultimately, these findings offer quantitative design guidelines for developing robotic manipulation systems with human‐like intuitive object perception and enhanced dexterity.

## 4 Experimental Section

### 4.1 Fabrication of Meissner Sensor

The fabrication of the Meissner sensor began by mixing the polydimethylsiloxane (PDMS, Dow Corning Sylgard 184) base and curing agent at a 10:1 weight ratio to obtain an uncured polymer mixture. The mixture was thoroughly stirred to ensure homogeneity for optimal curing. Carbon tape electrodes and polyvinylidene fluoride (PVDF) films (80 µm thickness) were precisely cut into the desired circular shapes using a laser cutter. The laser‐cut carbon tape electrodes were arranged in an orthogonal 90° configuration on the PVDF film to form the electrode structure of the Meissner‐like sensing layer (Figure S6). The prepared PDMS solution was then poured into a silicone mold designed to replicate dermal papillae‐like microstructures and cured in an oven at 60°C for 12 h to form a solid elastomeric structure. The cured PDMS exoskeleton was subsequently combined with the sensing layer fabricated in the previous step to assemble the integrated tactile sensor. To emulate the compliant dermal layer of human skin, Ecoflex 00–10 (produced by Smooth‐On, Inc) —an elastomer with a much lower elastic modulus than PDMS—was added between the structural and sensing layers. A silicone mold was fabricated by pouring Mold Max (produced by Smooth‐On, Inc.) silicone solution over a resin prototype produced via 3D printing and curing it at room temperature. The use of laser‐cut carbon electrodes and PVDF films facilitated accurate alignment and reliable integration within the sensor body (Figure S1A).

### 4.2 Fabrication of Merkel Sensor

We fabricated the pressure sensor by laser‐cutting a pressure‐sensitive film (3 m Velostat electrically conductive copolymer) to match the sensor dimensions (10 mm × 10 mm). The Velostat film was selected to obtain a stable and sustained piezoresistive response under normal pressure, reflecting the slowly adapting pressure‐sensing behavior of biological Merkel disks (Table S3). The laser‐cut film was then equipped with conductive thread electrodes arranged in a grid pattern on both sides. After securing the electrodes, polyimide tape was attached to both sides to ensure stable fixation (Figure S1B).

### 4.3 Fabrication of Ruffini Sensor

To mimic the nerve fiber structure within the Ruffini ending, we selected multi‐walled carbon nanotubes (MWCNTs, US Research Nanomaterials, Inc.) with a network structure as a piezoresistive material for fabricating a strain sensor. These sensing components were encapsulated in Ecoflex (elastic modulus E: ∼300 kPa), forming a soft capsule‐like layer inspired by the compliant encapsulating structure of biological Ruffini endings (Table S3).

First, we spin‐coated Ecoflex 00–30 onto a PTFE substrate at 300 rpm for approximately 60 s to ensure uniform distribution of the polymer across the substrate. Masking was then applied to define the sensor dimensions (10 mm × 6 mm), with the masking film prepared via laser cutting. Conductive electrodes were inserted and secured before the Ecoflex was fully cured. Next, MWCNTs were added to isopropyl alcohol (IPA, 99.0% purity) at a concentration of 0.03% and dispersed using bath sonication for 3 h to achieve uniformity. The resulting MWCNT solution was deposited onto the Ecoflex substrate via spray coating. Subsequently, Ecoflex was uniformly applied over the MWCNTs using a doctor‐blade technique. To enable infiltration of Ecoflex into the CNT network, the assembly was placed in a vacuum chamber for approximately 10 min. Finally, the structure was cured at room temperature for 4 h, resulting in a composite encapsulated artificial Ruffini sensor (Figure S1C).

### 4.4 Assembly of Artificial Tactile Sensors

The fabrication process for integrating individually fabricated shear, compression, and strain sensors was as follows: The sensors were aligned sequentially to match the structure of mechanoreceptors. Sil‐Poxy (produced by Smooth‐On, Inc) was used as an adhesive layer between the sensors. Subsequently, a packaging mold was designed to fit the dimensions of the integrated sensor. The packaging mold was 3D‐printed, filled with silicone, and cured to produce a flexible silicone mold. Next, the integrated sensor, assembled from the individual sensors, was placed into the mold, and a PDMS mixture (10:1 ratio) was poured in, cured at 80°C for 6 h, and then demolded (Figure S7). Finally, a high‐friction film (3 m 5401) was attached to the packaging surface to enhance durability and frictional force (Figure S5).

### 4.5 Readout Circuit

Each integrated sensor comprises one pair of carbon tape‐based electrodes for the Artificial Meissner sensor and two pairs of conductive thread‐based electrodes for the Artificial Merkel and Artificial Ruffini sensors. The Artificial Meissner sensor operates through a piezoelectric mechanism, and its voltage output was measured directly using an oscilloscope without a resistive voltage‐divider circuit, charge amplifier, or separate voltage amplifier.

The Artificial Merkel and Artificial Ruffini sensors are piezoresistive and were measured using a custom 25 mm × 25 mm four‐channel voltage‐divider PCB. In each channel, a 100 kΩ resistor and the connected sensor form a voltage divider, with the output voltage measured across the sensor. For one integrated sensor, the Merkel and Ruffini sensors were connected to the S1A and S1B input terminals, respectively, and the corresponding outputs were recorded using separate oscilloscope channels. Because each integrated sensor contains two piezoresistive sensing elements, one PCB can accommodate up to two integrated sensors. The PCB was powered by an external 9 V supply and included 10 and 0.1 µF decoupling capacitors and a 1N4007 diode for reverse‐polarity protection (Figure S27). All sensor signals were acquired without additional signal amplification.

### 4.6 Machine Learning and Ablation‐Based Mechanoreceptor Contribution Analysis

Tactile signals were collected from three mechanoreceptor‐inspired sensing channels: Meissner‐like shear, Merkel‐like pressure, and Ruffini‐like strain sensors. For machine‐learning‐based tactile classification, time‐domain statistical features, including maximum, minimum, mean, standard deviation, slope, and range, were extracted independently from each sensing channel. Before feature extraction, each signal was baseline‐corrected using the resting‐state signal measured before stimulation. All three sensing channels were acquired simultaneously using different channels of the same oscilloscope at an identical sampling rate within each experiment. Therefore, the Meissner‐like, Merkel‐like, and Ruffini‐like signals shared a common time axis.

A Random Forest classifier was used as the representative classifier in the main analysis. Random Forest‐based feature importance was used as a model‐specific supporting analysis to identify highly weighted tactile features. To quantify receptor‐level contribution more directly, channel‐ablation analysis was performed by removing each sensing channel and measuring the resulting decrease in classification performance. The Meissner‐like, Merkel‐like, and Ruffini‐like contributions were calculated by removing the shear, pressure, and strain feature groups, respectively, and comparing the resulting performance with that of the full three‐channel model.

To evaluate whether the ablation‐based contribution trends were dependent on the classifier, the same channel‐ablation procedure was additionally performed using multiple machine‐learning algorithms, including support vector machine (SVM), Gradient Boosting, multilayer perceptron (MLP) neural network, Logistic Regression, and k‐nearest neighbors (k‐NN), in addition to Random Forest. For all classifiers, the same feature set, data‐splitting strategy, cross‐validation procedure, and channel‐ablation method were applied. The multi‐classifier ablation results are summarized in Figure S23.

Model performance was evaluated using stratified k‐fold cross‐validation. Five folds were used unless the minimum class size was smaller than five, in which case the number of folds was adjusted according to the smallest class size. In each fold, approximately 80% of the data were used for training and 20% for testing. The data were shuffled before splitting, and a fixed random seed of 42 was used for reproducibility. For classifiers requiring feature scaling, including SVM, MLP, Logistic Regression, and k‐NN, standardization was performed within each cross‐validation fold using only the training data to avoid data leakage.

Extensive hyperparameter optimization was not performed because the purpose of this analysis was to evaluate whether the sensor‐contribution trends were robust across different classifiers, rather than to maximize the classification accuracy of each individual model. Therefore, fixed and commonly used hyperparameters were applied consistently across all tasks. The detailed classifier settings and validation conditions are summarized in Table S1.

## Author Contributions

Y. ‐J. Lee, J. Y. Woo, and C. ‐S. Han conceived the idea and designed the research. Y. ‐J. Lee developed the methodology. Y. ‐J. Lee and H. Kang carried out the device fabrication. Y. ‐J. Lee conducted the experimental investigation, performed the data curation and visualization, and designed the analysis. D. Kim designed the machine learning models and algorithms. C. ‐S. Han supervised the project and acquired the funding, and J. Y. Woo and C. ‐S. Han administered the project. Y. ‐J. Lee and C. ‐S. Han wrote the original draft of the manuscript. Y. ‐J. Lee, D. Kim, and C. ‐S. Han contributed to the writing, review, and editing of the manuscript.

## Funding

National Research Foundation of Korea (NRF) funded by the Ministry of Science and ICT (MSIT) grant RS‐2023‐00255584.

## Conflicts of Interest

The authors declare no conflicts of interest.

## Supporting information




**Supporting File 1**: advs77230‐sup‐0001‐SuppMat.docx.

**Supporting File 2**: advs77230‐sup‐0002‐MovieS1.mp4.

**Supporting File 3**: advs77230‐sup‐0003‐MovieS2.mp4.

**Supporting File 4**: advs77230‐sup‐0004‐MovieS3.mp4.

**Supporting File 5**: advs77230‐sup‐0005‐MovieS4.mp4.

**Supporting File 6**: advs77230‐sup‐0006‐MovieS5.mp4.

## Data Availability

The data that support the findings of this study are available from the corresponding author upon reasonable request.
