# Supplementary material for: A Bioinspired, Multimodal Soft Tactile Skin with Task‐Adaptive Perception for Intelligent Robotic Manipulation
Source: Adv Sci (Weinh). 2026 Aug 18:e77230. Online ahead of print. doi: 10.1002/advs.77230 (PMC13483134; doi:10.1002/advs.77230)
Supplement: Supplementary file 1 [file ADVS-9999-e77230-s001.docx]

Supporting Information

A Bioinspired, Multimodal Soft Tactile Skin with Task-Adaptive Perception for Intelligent Robotic Manipulation

Yu-Jin Lee^1,3^, Hyeonung Kang^1,3^, Ju Yeon Woo^3^*, Daekyum Kim^1,2^*, Chang-Soo Han^1,3^*

^1^ School of Mechanical Engineering, Korea University, Anam-Dong, Seongbuk-Gu, Seoul 02841, Rep. of Korea

^2^ School of Smart Mobility, Korea University, Anam-Dong, Seongbuk-Gu, Seoul 02841, Rep. of Korea

^3^ Somatosensory Molecular Biomimetics Research Center, Korea University, Anam-Dong, Seongbuk-Gu, Seoul 02841, Rep. of Korea

*Corresponding authors: cshan@korea.ac.kr, daekyum@korea.ac.kr, jywoo0780@korea.ac.kr

**This file includes:**

Figs. S1 to S27

Table S1 to S3

**Other Supplementary Materials for this manuscript include the following:**

Movie S1 to S5

**Fig. S1. Fabrication processes and photographs of individual artificial tactile sensors before integration.** (A) Schematic illustration of the fabrication process and photograph of the fabricated Artificial Meissner sensor. (B) Schematic illustration of the fabrication process and photograph of the fabricated Artificial Merkel sensor. (C) Schematic illustration of the fabrication process and photograph of the fabricated Artificial Ruffini sensor.

**Fig. S2. The sensing performance of Meissner sensor.** (A) Peak output voltage of the Meissner-like sensor as a function of vibration frequency. The output increased linearly in the 5–50 Hz range, exhibiting a frequency sensitivity of 0.679 mV/Hz (R^2^ = 0.952), and subsequently reached a nearly constant plateau at higher frequencies. (B) Sensitivity of the sensor under normal pressure, demonstrating relatively low responsiveness consistent with Meissner corpuscles’ physiological characteristics.

**Fig. S3. The sensing performance of Merkel sensor.** (A) Voltage responses of the Merkel sensor under normal pressures ranging from 0.5N to 7N. (B) Sensitivity of the sensor across two pressure ranges, showing ~0.06V/N below 6N and ~0.01V/N below 10N.

**Fig. S4. The sensing performance of Ruffini sensor.** (A) Characteristics of the Ruffini sensor under incremental uniaxial strain up to 30%. (B) Comparison of voltage responses under 10% tensile strain and under a normal pressure of 5 N, indicating that the sensor exhibits greater sensitivity to tensile strain than to normal pressure.

**Fig. S5. Fabrication of artificial integrated sensor.** Individual Ruffini, Merkel, and Meissner units were fabricated and vertically assembled according to their physiological distribution, with the Ruffini unit located at the deepest layer, the Merkel unit at the intermediate layer, and the Meissner unit at the uppermost layer.

**Fig. S6. Comparison of electrode routing strategies in vertically stacked sensors.** (A) Electrodes routed at 90° angles for vertical exit paths. (B) Electrodes extended laterally in a linear arrangement.

The 90° routing (A) enables a more compact integration by minimizing lateral space consumption in multilayer sensor configurations.

**Fig. S7. Packaging materials design for integrated sensors.** (A) Integrated sensor packaged with PDMS. (B) Integrated sensor packaged with Ecoflex 0030 (C) Resistance responses of the Ruffini sensor under different packaging materials. The enhanced response under Ecoflex indicates higher sensitivity to normal pressure, whereas PDMS suppresses such interference, making it more suitable for isolating strain-specific stimuli.

**Fig. S8. Prototype fabrication of the gripper hand.** (A) CAD design of the gripper hand for 3D printing. (B) Front view of the 3D-printed gripper hand with integrated sensors attached. (C) Back view showing the electrode routing of the integrated sensors on the gripper hand.

**Fig. S9. Lateral shear responses along the x- and y-directions.** (A) Schematic illustration of the lateral shear test. After establishing the same normal contact condition, the indenter was translated along either the x- or y-direction using the same lateral displacement and translation speed. (B) Representative responses of the artificial Meissner, Merkel, and Ruffini sensors under x- and y-direction shear loading.

The Meissner-like channel exhibited clear transient responses in both directions, while the Merkel-like channel showed comparable pressure-related responses. The Ruffini-like response was slightly smaller under y-direction shear because the strain-sensitive element was oriented along the x-axis and was therefore more sensitive to longitudinal than transverse deformation. These results demonstrate that the integrated sensor can detect shear-related events in both in-plane directions. However, because the present device comprises a single artificial Meissner sensing unit rather than a spatially distributed multi-axis sensor array, it was not designed to independently resolve the absolute direction of the applied shear force. Moreover, the present study focused on the rapid detection of shear-related dynamic events, such as incipient slip, rather than precise discrimination of the shear direction.

**Fig. S10. Response-time characterization of the artificial tactile sensing elements.** Representative transient voltage responses of the (A) Artificial Merkel sensor, (B) Artificial Meissner sensor, and (C) Artificial Ruffini sensor recorded at 10 kS/s. The Artificial Merkel sensor was tested under z-axis normal loading (~3 N), while the Artificial Meissner and Artificial Ruffini sensors were tested under x-axis lateral shear loading (~6 N). Response and release times were calculated from the 5–90% and 90–5% voltage changes, respectively.

**Fig. S11. Distinguishable multimodal responses under mixed rolling contact.** (A) Schematic of the rolling ball contact experiment, in which normal pressure, lateral shear, transient vibration, and tensile deformation were simultaneously generated. (B) Representative vertically offset voltage responses of the three sensing channels during rolling contact. (C) Inter-channel Pearson correlation matrix of the baseline-subtracted signals, used to evaluate signal similarity under mixed mechanical loading. (D) Dynamic-band energy percentage of each channel. The 5–50 Hz band was selected as the Meissner-sensitive dynamic band associated with low-frequency flutter/vibration, and the percentage was calculated relative to the total spectral energy within the 0.5–50 Hz analysis band. The Meissner-like channel exhibited the highest dynamic-band energy percentage, indicating that rolling-induced shear/vibration components were predominantly captured by the Meissner-like sensing unit.

**Fig. S12. Normalized stimulus–response matrix of the integrated tactile sensor under controlled single-input loading conditions.** The responses of the Merkel-, Meissner-, and Ruffini-like sensing channels were evaluated under normal-pressure-only, lateral-shear-only, and tensile-strain-only loading. After baseline subtraction, the Merkel- and Ruffini-like responses were quantified using the absolute steady-state amplitude, reflecting their slowly adapting characteristics, whereas the Meissner-like response was quantified using the dynamic root-mean-square amplitude within the designated loading interval, reflecting its rapidly adapting behavior. For each sensing channel, the responses were normalized to the amplitude obtained under its preferred stimulus, defined as 100%: normal pressure for the Merkel-like channel, lateral shear for the Meissner-like channel, and tensile strain for the Ruffini-like channel. The strong diagonal dominance of the matrix demonstrates preferential stimulus selectivity of the three sensing channels. Limited cross-responses were observed mainly in the Merkel-like channel under shear and tensile loading, which were attributed to secondary compression or bending mechanically transferred through the integrated soft structure rather than electrical cross-talk.

**Fig. S13. Experimental setup for braille reading.** (A) Integrated tactile sensor attached to the fingertip. (B) Photograph of the Braille reading process using a 3D-printed Braille sample.

**Fig. S14. Experimental setup for texture recognition.** (A) Experimental setup with a linear motor stage for applying lateral scanning motion across textured surfaces. (B) Signal outputs obtained from three consecutive scanning motions (right–left–right). The left graph shows responses for the bubble wrap sample, while the right graph shows responses for the glass sample. A clear difference is observed in the Ruffini sensor output, attributed to greater surface-induced stretch from the higher friction of the glass sample.

**Fig. S15. Experimental setup for slip.** (A) Initial gripping state using a two-finger robotic gripper without an attached weight. (B) Induced slippage by attaching a weight to the vial.

**Fig. S16. Grasping and slip responses for different vial surface materials.** (A) Schematic illustration of the vial-surface materials used in the grasping experiment, including bare glass, stainless-steel mesh, and corrugated cardboard. The background colors indicate the grasping state: light green represents the stable grip state, whereas light purple represents the slip/drop state. (B–D) Representative responses of the integrated tactile sensor during grasping and slip tests with different vial-surface materials: (B) bare glass, (C) stainless-steel mesh, and (D) corrugated cardboard.

For bare glass, the Meissner-like channel exhibited relatively small fluctuations during sliding but distinct transient peaks at slip onset and object release, while the Ruffini-like channel showed a comparatively large response associated with lateral deformation of the integrated sensor surface. For the stainless-steel mesh, the periodic grid structure generated repeated shear variations and small interfacial vibrations, producing oscillatory responses in the Meissner-like channel during sliding. For corrugated cardboard, the larger and more pronounced surface corrugations caused greater sliding instability and repeated shear- and vibration-related fluctuations, resulting in the most prominent dynamic response in the Meissner-like channel, whereas the Merkel- and Ruffini-like channels exhibited comparatively smaller changes.

**Fig. S17. Experimental setup for softness test.** Softness testing using an Ecoflex 0010 sample with the integrated tactile sensor attached to the probe.

**Fig. S18. Random forest–based confusion matrices for sensor combinations in braille reading.** Braille letter classification for six characters (“T,” “A,” “C,” “T,” “I,” “L,” “E”) using single- and multimodal tactile cues. Results are shown for (A) strain, (B) pressure, and (C) shear sensors alone, and (D) strain + pressure, (E) pressure + shear, and (F) strain + shear combinations.

**Fig. S19. Random forest–based confusion matrices for sensor combinations in texture classification.** Texture classification across ten materials (bubble wrap, corrugated cardboard, fabric, foam, glass, nonwoven, paper, rubber, sandpaper, and stainless steel mesh) using single- and multimodal tactile sensing. Results are shown for (A) strain, (B) pressure, and (C) shear sensors alone, and (D) strain + pressure, (E) pressure + shear, and (F) strain + shear combinations. Shear sensing (C) yields superior performance among single modalities, underscoring the importance of lateral tactile cues in surface discrimination.

**Fig. S20. Random forest–based confusion matrices for sensor combinations in slip detection.** Classification performance for three interaction states—normal contact, slip, and object drop—using single- and multimodal tactile sensing. Results are shown for (A) strain, (B) pressure, and (C) shear sensors alone, and (D) strain + pressure, (E) pressure + shear, and (F) strain + shear combinations.

**Fig. S21. Random forest–based confusion matrices for sensor combinations in softness classification.** Classification results for five softness levels using objects of varying compliance: ball, Ecoflex 0010, Ecoflex 0030, Ecoflex 0050, and PDMS (20:1). Performance is shown for (A) strain, (B) pressure, and (C) shear sensing alone, and multimodal combinations of (D) strain + pressure, (E) pressure + shear, and (F) strain + shear.

**Fig. S22. Random forest–based confusion matrices from combined sensors.** (A) Texture recognition (B) Braille reading (C) Slip detection (D) Softness recognition

**Fig. S23. Classifier-independent ablation contribution analysis.** Ablation contribution of the Meissner-like shear, Merkel-like pressure, and Ruffini-like strain channels for (A) Braille recognition, (B) surface texture recognition, (C) slip/drop detection, and (D) softness recognition. Contribution was defined as the decrease in classification performance after removing each sensing channel from the full three-channel model. A larger value indicates a greater contribution of the removed channel. The analysis was repeated using Random Forest, SVM, Gradient Boosting, MLP neural network, Logistic Regression, and k-NN. The results show task-dependent dominant sensing channels: Merkel-like pressure for Braille recognition, Meissner-like shear for texture recognition and slip/drop detection, and Ruffini-like strain/Merkel-like pressure for softness recognition.

**Fig. S24. Experimental setup and mechanoreceptor responses during vial manipulation.** (A) Hand grasping a 30ml vial with integrated sensor attached to the fingers. (B) Lift-and-hold phase following initial grasp. (C) Representative responses of mechanoreceptors (Merkel, Meissner, Ruffini) throughout the grasp-lift-hold-release sequence, illustrating their distinct activation patterns across each phase.

**Fig. S25. Signal responses of the integrated tactile sensor during the surface-defect detection procedure performed on a defect-free automotive surface.**

**Fig. S26. Integrated sensor responses to graded abrasive surfaces.** (A) Sensor output profiles in response to sandpaper samples of increasing surface roughness (P2500 to P60); voltage amplitudes increase with grit coarseness, indicating graded texture encoding. (B) Schematic of the experimental setup for surface interaction; table summarizes the full set of abrasive samples (P2500, P1200, P600, P240, P150, P100, P60) used in the texture classification task.

**Fig. S27. Readout configurations for piezoelectric and piezoresistive sensing units.** (A) Photograph and circuit schematic of the custom PCB used for piezoresistive sensor readout. (B) Signal acquisition schematic of the Artificial Meissner sensor. The piezoelectric voltage generated from the PVDF sensing layer was measured directly using an oscilloscope without a resistive voltage-divider circuit, charge amplifier, or separate voltage amplifier. (C) Signal acquisition schematic of the Artificial Merkel and Artificial Ruffini sensors. The resistance changes of the piezoresistive sensors were converted into voltage outputs using the custom voltage-divider PCB and recorded using separate oscilloscope channels. All sensor signals were acquired without additional signal amplification.

**Table S1. Classifier settings used for the multi-classifier ablation analysis.**

| Classifier | Model type | Main hyperparameters | Feature scaling |
| --- | --- | --- | --- |
| Random Forest | Tree-based ensemble | 300 trees; square-root feature sampling; minimum samples per leaf=2 | Not applied |
| SVM | Margin-based classifier | RBF kernel; C=1.0; gamma=scale | StandardScaler |
| Gradient Boosting | Tree-based ensemble | 50 estimators; learning rate=0.05; max depth=1; subsample=0.7; minimum samples per leaf=3 | Not applied |
| MLP neural network | Nonlinear neural-network classifier | Three hidden layers with 128, 64, and 32 neurons; ReLU activation for hidden layers; linear output layer; Adam optimizer; learning rate = 0.001; L2 regularization coefficient = 0.001; maximum epochs = 400; early-stopping patience = 50 | StandardScaler |
| Logistic Regression | Linear baseline classifier | L2 regularization; C=1.0; lbfgs solver; maximum iteration=3000 | StandardScaler |
| k-NN | Distance-based non-parametric classifier | K=7; uniform weighting; Euclidean distance | StandardScaler |

All classifiers were evaluated using stratified k-fold cross-validation with shuffling and a fixed random seed of 42. Five folds were used unless the minimum class size was smaller than five, in which case the number of folds was adjusted according to the smallest class size. For classifiers requiring feature scaling, StandardScaler was fitted only on the training data within each fold and then applied to the corresponding test data to avoid data leakage. Extensive hyperparameter optimization was not performed; fixed hyperparameters were used to compare the robustness of sensor-contribution trends across classifiers.

**Table S2. Functional comparison between biological Meissner corpuscles and the Artificial Meissner sensor**

**Table S3. Comparison of the functional characteristics of biological Merkel disks and Ruffini endings with the corresponding artificial sensors**

**References**

1. Ikeda, Ryo, et al., “Merkel cells transduce and encode tactile stimuli to drive Aβ-afferent impulses,” *Cell* 157.3 (2014): 664-675.

<https://doi.org/10.1016/j.cell.2014.02.026>

2. Woo, Seung-Hyun, et al., “Piezo2 is required for Merkel-cell mechanotransduction,” *Nature* 509.7502 (2014): 622-626.

<https://doi.org/10.1038/nature13251>

3. Chambers, Margaret R., et al., “The structure and function of the slowly adapting type II mechanoreceptor in hairy skin,” *Quarterly Journal of Experimental Physiology and Cognate Medical Sciences: Translation and Integration* 57.4 (1972): 417-445.

<https://doi.org/10.1113/expphysiol.1972.sp002177>

4. Cobo, Ramón, et al., “Verification and characterisation of human digital Ruffini’s sensory corpuscles,” *Journal of Anatomy* 238.1 (2021): 13-19.

<https://doi.org/10.1111/joa.13301>

5. Watkins, Roger Holmes, et al., “Slowly‐adapting type II afferents contribute to conscious touch sensation in humans: Evidence from single unit intraneural microstimulation,” *The Journal of Physiology* 600.12 (2022): 2939-2952.

<https://doi.org/10.1113/JP282873>

6. Johansson, R. S., Å. B. Vallbo, and G. Westling., “Thresholds of mechanosensitive afferents in the human hand as measured with von Frey hairs,” *Brain research* 184.2 (1980): 343-351.

<https://doi.org/10.1016/0006-8993(80)90803-3>

7. García-Mesa, Y., García-Piqueras, J. et al., “Merkel cells and Meissner's corpuscles in human digital skin display Piezo2 immunoreactivity,” *Journal of Anatomy* 231.6 (2017): 978-989.

<https://doi.org/10.1111/joa.12688>

8. Purali, N., “Mechanosensitive Ion Channels: The Unending Riddle of Mechanotransduction,” Bioelectricity, 7.1 (2024): 58-70.

<https://doi.org/10.1089/bioe.2024.0028>

9. Handler, Annie, and David D. Ginty., “The mechanosensory neurons of touch and their mechanisms of activation,” *Nature Reviews Neuroscience* 22.9 (2021): 521-537.

<https://doi.org/10.1038/s41583-021-00489-x>

10. Johansson, Roland S., and Ake B. Vallbo., “Tactile sensibility in the human hand: relative and absolute densities of four types of mechanoreceptive units in glabrous skin,” *The Journal of physiology* 286.1 (1979): 283-300.

<https://doi.org/10.1113/jphysiol.1979.sp012619>
